# Expanding the limits of synthetic macromolecular chemistry through Polyphenylene Dendrimers

**DOI:** 10.1007/s11051-018-4364-6

**Published:** 2018-09-25

**Authors:** Brenton A. G. Hammer, Klaus Müllen

**Affiliations:** 1 Department of Chemistry and Biochemistry, California State University Northridge, 18111 Nordhoff St. 91330, Northridge, CA USA; 2 Max Planck Institute for Polymer Research, Ackermannweg 10, 55128 Mainz, Germany

**Keywords:** Polyphenylene dendrimers, Dendritic properties, Supramolecular interactions, Complex architectures, Macromolecular chemistry, Synthesis methods

## Abstract

Polyphenylene dendrimers (PPDs) are a unique class of macromolecules because their backbone is made from twisted benzene repeat units that result in a rigid, shape-persistent architecture as reported by Hammer et al. (Chem Soc Rev 44:4072–4090, 2015) and Hammer and Müllen (Chem Rev 116:2103–210, 2016) These dendrimers can be synthetically tailored at their core, scaffold, and surface to introduce a wide range of chemical functionalities that influence their applications. It is the balance between the macromolecular properties of polyphenylene dendrimers with grandiose synthetic ingenuity that presents a template for the next generation of synthetic dendrimers to achieve complex structures other chemistry fields cannot. This perspective will look at how advances in synthetic chemistry have led to an explosion in the properties of polyphenylene dendrimers from their initial stage, as PPDs that were used as precursors for nanographenes, to next-generation dendrimers for organic electronic devices, sensors for volatile organic compounds (VOCs), nanocarriers for small molecules, and even as complexes with therapeutic drugs and viruses, among others. Ideally, this perspective will illustrate how the evolution of synthetic chemistry has influenced the possible structures and properties of PPDs and how these chemical modifications have opened the door to unprecedented applications.

## Introduction

Polyphenylene dendrimers (PPDs) are highly branched, monodisperse macromolecules that are shape-persistent because their backbone consists of substituted benzene rings (Feng et al. 2009; Hammer and Müllen 2016; Imai and Arai 2002; Shen et al. 2004; Stangenberg et al. 2014a, b; Türp et al. 2012; Wiesler et al. 2001). Thus, these dendrimers are extremely stable and can be modified in a site-specific manner since their dendrons cannot reorient due to the rigidity of the backbone (Baumgarten, 2015). PPDs have three levels: the core, scaffold, and surface, where it is possible to synthetically tailor the functionalities at all three stages. It is this synthetic versatility that has led to the evolution in the applications for PPDs from nanographenes to complex macromolecules that have been incorporated into organic electronics, used as weakly coordinating anions and cations and even mimicked biologically relevant proteins, among many other applications (Hammer et al. 2015; Müller and Müllen 2007). The hope of this perspective is to outline the advantages and achievements of PPDs as compared to other hyperbranched and dendritic materials and to encourage the next wave of synthetic achievements in the field.

**Fig. 1:**
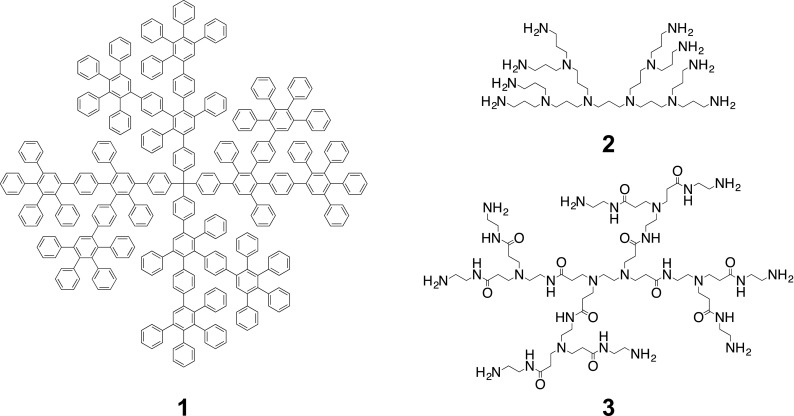
Chemical structures of a second-generation PPD, PPI, and PAMAM dendrimer

It is important to note the differences between polyphenylene dendrimers and other dendrimer families such as poly(amidoamide) (PAMAM) and poly(propylene imine) (PPI), as it is these differences that allow PPDs to be synthesized and applied in such unique ways. One of the most important aspects lies in the shape-persistent nature of PPDs. Figure 1 shows the chemical structure of a second-generation PPD (1), PPI (2), and PAMAM (3). The PPD consists of a rigid phenylene-based backbone, while PAMAM and PPI have flexible aliphatic backbones. This difference in the rigidity of the backbones explains why PPDs are shape-persistent, and can thus be synthesized with nano-site precision, as compared to some other dendrimers that can undergo conformational rearrangements leading to a structural equilibrium of where chemical functionalities are located. This reduced conformational flexibility is a key aspect when considering chemically modifying the core, scaffold, and surface of a dendrimer, because PPDs can be functionalized at any of those levels in a site-specific manner and their exact location is known, and, thus, the targeted properties of the macromolecules (Hammer and Müllen 2016; Narita et al. 2015; Watson et al. 2001).

In general, dendrimers are classified as being “core dense” because their dendritic arms consist of flexible molecules that adopt a random coil orientation which can lead to their collapsing around the core (Astruc 2001; Newkome and Shreiner 2008; Maraval et al. 2003; Rosenfeldt et al. 2005; Tomalia et al. 1990). Such properties can be a disadvantage if specific chemical functionalities are supposed to be on the exterior of a dendrimer, as they are actually engulfed within the interior of the structure. This is where the shape persistence of PPDs and their “semi-rigid” dendrons is advantageous, because they cannot undergo such conformational rearrangements. Thus, without the possibility of back bending of dendron arms, the surface moieties are always on the outside of the dendrimers. PPDs are rightfully classified as “surface dense” dendrimers with defined interior cavities (Bieri et al. 2010; Wind et al. 2002) As the dendrimers are synthesized to higher generations (i.e., larger molecules) the surface-bound phenylene groups grow closer together and become sterically congested which leads to a globular shape. The shape-persistent, globular structure of PPDs has been confirmed by AFM, TEM, computer simulations, and solid-state NMR spectroscopy. Doing solid-state NMR spectroscopy with rapid magic angle spinning illustrated the restricted rotation of the interior versus exterior phenyl rings in the MHz regime, where the terminal rings had fast vibrations as compared to the slow dynamics of the core and scaffold phenylene groups due to restricted movement (Wind et al. 2002). AFM and TEM images of polyphenylene dendrimers show globular structures based on the generation, and the overall shape was dependent on the geometry which is determined by the chosen dendrimer core (Stangenberg et al. 2014a, b; Zhang et al. 2000; Brocorens et al. 2007; Carbone et al. 2007; Melnikov et al. 2007; Rosenfeldt et al. 2005; Wind et al. 2000). X-ray structural analysis of polyphenylene dendrimers showed their single-crystal structure to be a network of rigid, twisted phenylene rings and open voids between the dendrons in the macromolecular interior, while the surface density increases at higher generations (Pisula et al. 2007). Furthermore, small-angle neutron scattering (SANS) was performed on a fourth-generation PPD synthesized from a biphenyl core and it displayed a “shell dense” architecture that was attributed to the rigidity of the phenylene backbone. Once again, this is in stark contrast to most dendrimers, such as PAMAM and PPI, because those macromolecules can undergo conformational rearrangements of their flexible arms and are therefore considered core dense materials (Fre 2002; Gillies and Fréchet 2005; Grayson and Fre 2001; Kannan et al. 2014; Rosenfeldt et al. 2005; Ruiz et al. 2003).

The versatility in the synthesis of polyphenylene dendrimers combined with their monodisperse, shape-persistent, and structural perfection opens the door to unprecedented applications. This perspective will provide an overview of complex syntheses of various PPDs at their core, scaffold, and surface with a correlation of how these modifications influence their bulk properties. Ideally, a story will develop between how state-of-the-art dendrimer syntheses have led to unprecedented applications ranging from organic electronic active layers, functional nanographenes, biomimicking macromolecules, nanocarriers for therapeutic drugs, and many more.

## Synthesis and functionalization of polyphenylene dendrimers

The unique shape-persistent, monodisperse nature of polyphenylene dendrimers is derived from their synthesis consisting of a core, whose structure dictates the number of arms and geometry of the dendrimer, and building blocks, which control the chemical functionalization of the scaffold and surface, as well as the generation of the macromolecule (i.e., dendrimer size) (Li et al. 2010; Wiesler et al. 2001; Zhang et al. 2014a, b). There are two synthetic pathways to form PPDs: a convergent and divergent approach. Convergent methods usually use a transition-metal cross-coupling reaction between a halide-functionalized aromatic core and dendrons possessing either a halide or boronate species; however, these reactions are not always quantitative so nearly all PPDs are synthesized via the divergent growth mechanism. In this case, a [4 + 2] Diels-Alder cycloaddition reaction between an ethynyl modified core and building blocks consisting of cyclopentadienone groups takes place to grow the dendrimer from the inside out (Morgenroth et al. 1997; Qin et al. 2009; Wiesler and Müllen 1999; Wiesler et al. 2001). This is significant not only in producing monodisperse macromolecules but also in that the only requirement for the reaction is to have a diene and dienophile that are stable at elevated temperatures (i.e., ~ 135–170 °C). The synthetic versatility enables the incorporation of numerous chemical functionalities at the core, scaffold, and/or on the surface of PPDs in a site-specific manner. Additionally, the Diels-Alder reaction is quantitative with irreversible conversions meaning the product is stable (Andreitchenko et al. 2008; Nguyen et al. 2013a, b). In contrast, some dendrimer families have flexible arms that can limit access to the growing chain ends due to conformational rearrangements and utilize a growth mechanism that is reversible. This synthetic approach may lead to an equilibrium of products, which limits quantitative synthesis and forms defects (Caminade and Majoral 2005; Hernandez-Lopez et al. 2003; Jansen et al. 1994; Newkome and Shreiner 2008; Maraval et al. 2003; Tomalia et al. 1990). Therefore, the synthetic strategy and shape-persistent nature of PPDs may be more likely to produce monodisperse, defect-free dendrimers with more predictable properties.

**Fig. 2:**
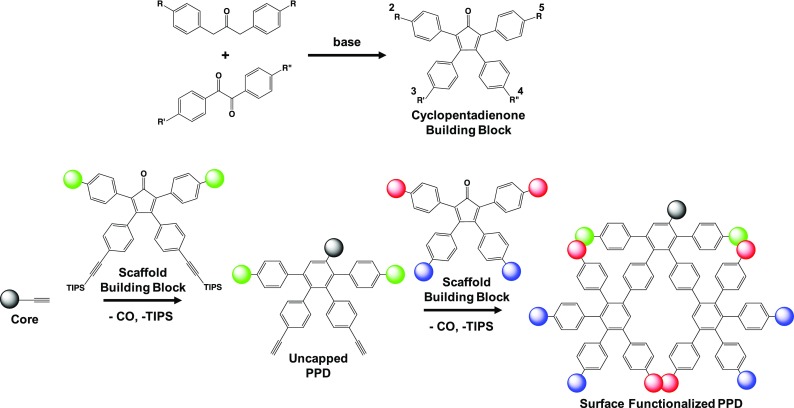
General synthetic scheme for PPDs

Figure 2 outlines the general synthesis for PPDs starting with a core (gray sphere), scaffold building blocks (green spheres), and surface building blocks (blue and red spheres). A core molecule is chosen based on the desired chemical functionality of the dendrimer center, and the geometry of the macromolecule (i.e., number and relative orientation of the arms). As previously mentioned, the only requirement for a core is that it has free ethynyl groups (dienophile) and is stable at elevated temperatures so a wide variety of molecules can be introduced into the dendrimer core.

**Fig. 3:**
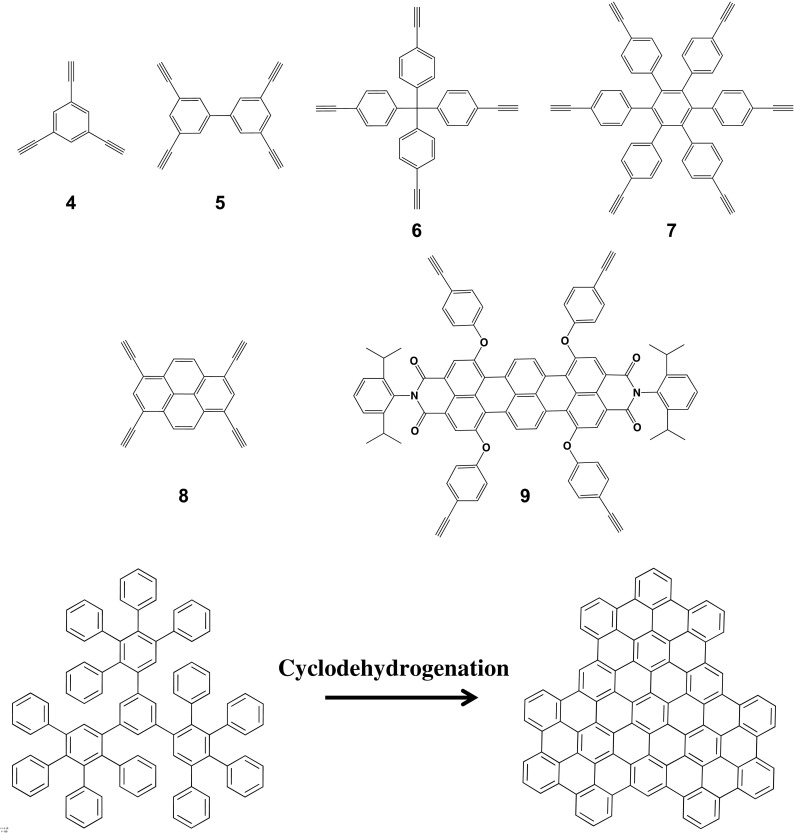
Commonly used core molecules for PPD syntheses and cyclodehydrogenation of a PPD to form a nanographene derivative

The scaffold building blocks (green spheres) are used to modify the interior cavity of PPDs (i.e., their scaffold), and they utilize a cyclopentadienone structure (diene). These cyclopentadienone molecules undergo the [4 + 2] Diels-Alder cycloaddition with the core to form a new benzene ring and the first generation of the dendrimer, and extrude carbon monoxide as a by-product. Typically, the chemical moieties at the 2- and 5-phenyl positions introduce hydrophobic or hydrophilic properties to the scaffold, and the 3- and 4-phenyl moieties have triisopropylsilyl (TIPS) ethynyl groups. The TIPS can be deprotected upon exposure to tetrabutyl ammonium fluoride (TBAF) to yield free ethynyl groups that can undergo subsequent cycloaddition to grow the dendrimers. These scaffold building blocks are classified as AB_2_ monomers because they turn one ethynyl into two new ethynyl groups after the initial Diels-Alder reaction and TIPS deprotection. AB_4_ scaffold building blocks can also be synthesized where TIPS ethynyl functionalities are at the 2-, 3-, 4-, and 5-phenyl positions, where deprotection with TBAF yields four free ethynyl groups to grow the next generation. Thus, they are named AB_4_ monomers for their ability to convert a single ethynyl moiety to four. Surface building blocks (blue and red spheres) use the same cyclopentadienone structure, but they are chemically modified on the 3- and 4-phenyl groups, which influence the surface properties of the PPDs. Once again, the only requirement for the building blocks is to have a cylcopentadienone base and be stable at elevated temperatures and this synthetic flexibility has allowed for the integration of a wide-range of chemical functionalities into the dendrimer core, scaffold, or onto its surface. Additionally, it is important to look at the general synthesis of a cyclopentadienone, which utilizes a Knoevenagel condensation of a diphenyl acetone- and benzyl-based molecules, since this is the step where the building blocks are chemically modified. One can synthesize homogenous PPDs if *R*′ and *R*″ are the same functionality, while unsymmetric dendrimers can be achieved by varying *R′* and *R″*. For example, cyclopentadienones were synthesized with a phenyl or ethynyl functionalized phenyl group at the 3-position and perylenedicarboximide (PMI) at the 4-position. This molecule underwent sequential Diels-Alder cycloaddition reactions from a terrylenetetracarbondiimide (TDI) core (Fig. 3, molecule 9) to form an unsymmetrical PPD (Grimsdale et al. 2002). These dendrimers display vectorial energy transfer from the surface to the core dye and their properties will be further discussed in section “What is on the outside matters.”

While this perspective focuses on polyphenylene dendrimers, it is critical to note their analogous hyperbranched polyphenylene counterparts. While their molecular make-up is very similar as they both are composed of a polyphenylene backbone, there are structural differences between the families that significantly influence their structural properties. Hyperbranched polyphenylenes are synthesized from AB_2_ monomers that typically utilize either palladium or nickel catalyzed aryl-aryl cross-coupling (Suzuki or Yamamoto) or Diels-Alder [4 + 2] cycloadditions (Morgenroth and Müllen 1997; “Water-Soluble Hyperbranched Polyphenylene,” n.d.; Zhi et al. 2005). These macromolecules typically have a high polydispersity index (> 2) which leads to defects and a lack of structural/chemical control, as compared to PPDs that are synthesized via sequential Diels-Alder cycloaddition that are monodisperse macromolecules (defect-free) with defined structures (Morgenroth et al. 1997; Morgenroth and Müllen 1997; Maraval et al. 2003; Morgenroth 1998; Water-soluble hyperbranched polyphenylene, n.d). Furthermore, hyperbranched polyphenylenes have been synthesized and cross-linked with 1,3,5-tris(azidomethyl) benzene to stabilize their morphologies, which is a necessity for their use as low-dielectric matrix material in microelectronics (Pötzsch and Voit 2012). Despite any imperfections of hyperbranched polyphenylenes, they have successfully been used in light-emitting devices, as low dielectric materials, and in self-assembly applications. However, the monodisperse, shape-persistent and stable qualities of PPDs have led to more sophisticated macromolecules which have been introduced into applications ranging from pristine light emitting devices to chemical sensors and even as nanocarriers for therapeutic drugs (Nguyen et al. 2013a, b; Türp et al. 2012).

## It all starts with the core

A core molecule is chosen based on the desired number and relative orientation of the dendron arms, and while the species must be stable to the elevated temperatures of a Diels-Alder cycloaddition reaction, there is extensive flexibility when choosing such a compound. Figure 3 illustrates many commonly used core functionalities which are normally benzene-based motifs, such as benzene-(4), biphenyl-(5), tetraphenylmethane-(6), and hexaphenylbenzene-based molecules (7) (Andreitchenko et al. 2008; Mihov et al. 2005; Wiesler et al. 2001). Each molecule has a different number of arms and geometry around the central atom, so which molecule is used depends on the desired shape, surface density, and steric access to the scaffold for the final dendrimer. In the mid-1990s, such molecules were used to synthesize PPDs that were converted to the first reported nanographenes (Bieri et al. 2009; Müller and Müllen 2007; Narita et al. 2015). Figure 3 shows the dendrimer product of core 3 undergoing a threefold Diels-Alder cycloaddition with 2,3,4,5-tetraphenylcyclopentadienone and subsequent oxidative cyclodehydrogenation combined with planarization to a nanographene (Andreitchenko et al. 2008; Wu et al. 2003; Blankenburg et al. 2012). Polyphenylene dendrimers were the first reported precursors for synthetic graphene materials where their dimensions could be controlled through the geometry and size (generation) of the PPDs, leading to unprecedented control of synthetic graphene-based macromolecules, which will be further covered in section “Looking Forward” (Angelova et al. 2013; Dössel et al. 2011; El Hamaoui et al. 2007; Müller and Müllen 2007; Narita et al. 2013, 2015; Rao et al. 2014; Schlütter et al. 2014; Wu et al. 2005)

In addition to simple benzene-based molecules, pyrene (8) and terrylenetetracarbondiimide (TDI) (9) have been introduced into the core of PPDs to control their optical properties. A second-generation dendrimer was synthesized from a pyrene core that incorporated four triphenylene units within the scaffold and had 16 triphenylamines (TPA) attached to the surface, in hopes of promoting hole capturing and injection of the TPA units and resonant transfer of the excitation energy from the triphenylenes towards the pyrene core. These dendrimers were used in an organic light-emitting diode (OLED) and their multilayer structure led to a highly stable blue emission with a brightness of 1440 cd/m^2^ (Bernhardt et al. 2005, 2006; Zhang et al. 2014a, b; Zöphel et al. 2013). In another case, a first-generation PPD that had a pyrene core was synthesized with different aryl-amine groups (diphenylamine, phenylnaphthylamine, etc.). These macromolecules also had a layer-by-layer design to encourage energy transfer from the surface to the pyrene center and achieved a pure blue emission with CIE coordinates of (0.16, 0.21) and a luminance as high as 3700 cd/m^2^. A TDI core was used to synthesize a third-generation dendrimer that had perylenedicarboximdes (PMI) bond to the scaffold and naphthalenedicarboximide (NMI) groups attached to the surface (Grimsdale et al. 2002; Gronheid et al. 2002; Herrmann et al. 2001; Métivier et al. 2004; Minard-Basquin et al. 2003; Weil et al. 2001). This design intended to use a stepwise vectorial energy transfer from the surface NMI units through the interior PMI moieties towards the TDI core and resulted in a light harvesting dendrimer that displayed absorption over the whole visible spectrum and little leaching of the excitation energy.

Traditionally, polyphenylene dendrimers were synthesized from organic core molecules; however, it is also possible to build them around transition-metal species through terminal ligation. Utilizing cyclic metallophthalocyanine functionalized dendrons led to the introduction of cobalt into the dendrimer core. The four dendrons selectively shielded the cobalt limiting its axial coordination for pyridine derivatives of various sizes, while the cobalt core improved the solubility of the PPDs in polar solvents (Kimura et al. 2003; Waybright et al. 2002). The optical properties of these dendrimers fluctuated upon interactions with gaseous pyridine-based molecules making them options for chemosensors. Alternatively, cobalt was functionalized with cyclopentadienyl (dicarbonyl) groups with available ethynyl end moieties, and these groups were reacted via Diels-Alder cycloaddition reaction with cyclopentadienones to form first-generation PPDs. In a similar approach, the four dendrons shielded the core cobalt to stabilize its electro-active 17 electron state, which led to an increased oxidation potential of 0.83 V, which also imparted stability of the active metal to air and water.

In a different approach, iridium (III) ions were inserted into a dendrimer core to manipulate its stability and optical properties (Qin et al. 2009, 2012), Fac-tris(2-benzo [b]thiophenylpyridyl)-based dendrons were synthesized and coordinated to an iridium(III) species, and first- through third-generation dendrimers were made from free surface-bound ethynyl groups. The surface of these PPDs was modified with triphenyl amines and these multi-layered dendrimers showed absorptions between 300 and 350 nm, an emission maximum at ~ 624 nm, and CIE coordinates of (0.63–0.68, 0.32) depending on the generation. These values indicate a pure red emission as referenced against the National Television System Committee standard for red subpixels (0.67, 0.33), and therefore viable options for OLED devices.

Utilizing polyphenylene dendrons as ligands can influence more than just the optical or catalytic properties of transition metals, it can also open the door for more complex architectures. One instance was when polyphenylene dendrons were functionalized with bipyridine ligands that underwent a ligand exchange with 4,4-bis (TIPS-ethynyl)-2,2′-bipyridine units coordinated to a cationic ruthenium complex. Based on the extent of ligand exchange, it was possible to achieve up to an octahedral geometry around the ruthenium core, while the dendrons were built up to the third generation (Grimes et al. 2010; Haberecht et al. 2008). This process produced large, asymmetric Ru cations with an ability to manipulate their geometry (octahedral), symmetry around the catalytic core, and, thus, the reactivity of the ruthenium ion, which had not been previously observed.

As synthetic techniques and objectives have evolved, so too have the desired properties of polyphenylene dendrimers based on more complex chemical structures. One of the more unique examples is in the field of weakly coordinating ions (WCIs) where either an anion (boron) (Türp et al. 2011) or cation (phosphorus) (Moritz et al. 2014) was inserted as the dendrimer core and PPDs up to the third generation were synthesized around them. WCIs are extremely appealing as materials for ionic liquids, electrochemistry, catalysis, and battery applications. Conceptually the large, rigid dendritic arms shield the Coulombic forces between associative ion pairs (i.e., the core and a counter ion) which allows for controlling the distance between the two species. By tuning the size (i.e., generation) of the cationic phosphorus- and anionic boron-based species and mixing them in the solution, it was possible to determine the Bjerrum length (λ_B_), the characteristic separation distance between two ions at which Coulombic interactions are balanced by thermal energy (Moritz et al. 2014). Additionally, it was possible to dissolve these cationic and anionic salts in unpolar solvents (hexane, diethyl ether, toluene) based on the hydrophobic nature of the shielding polyphenylene dendrons, which represent a unique class of WCIs that have a range of solubilities.

**Fig. 4:**
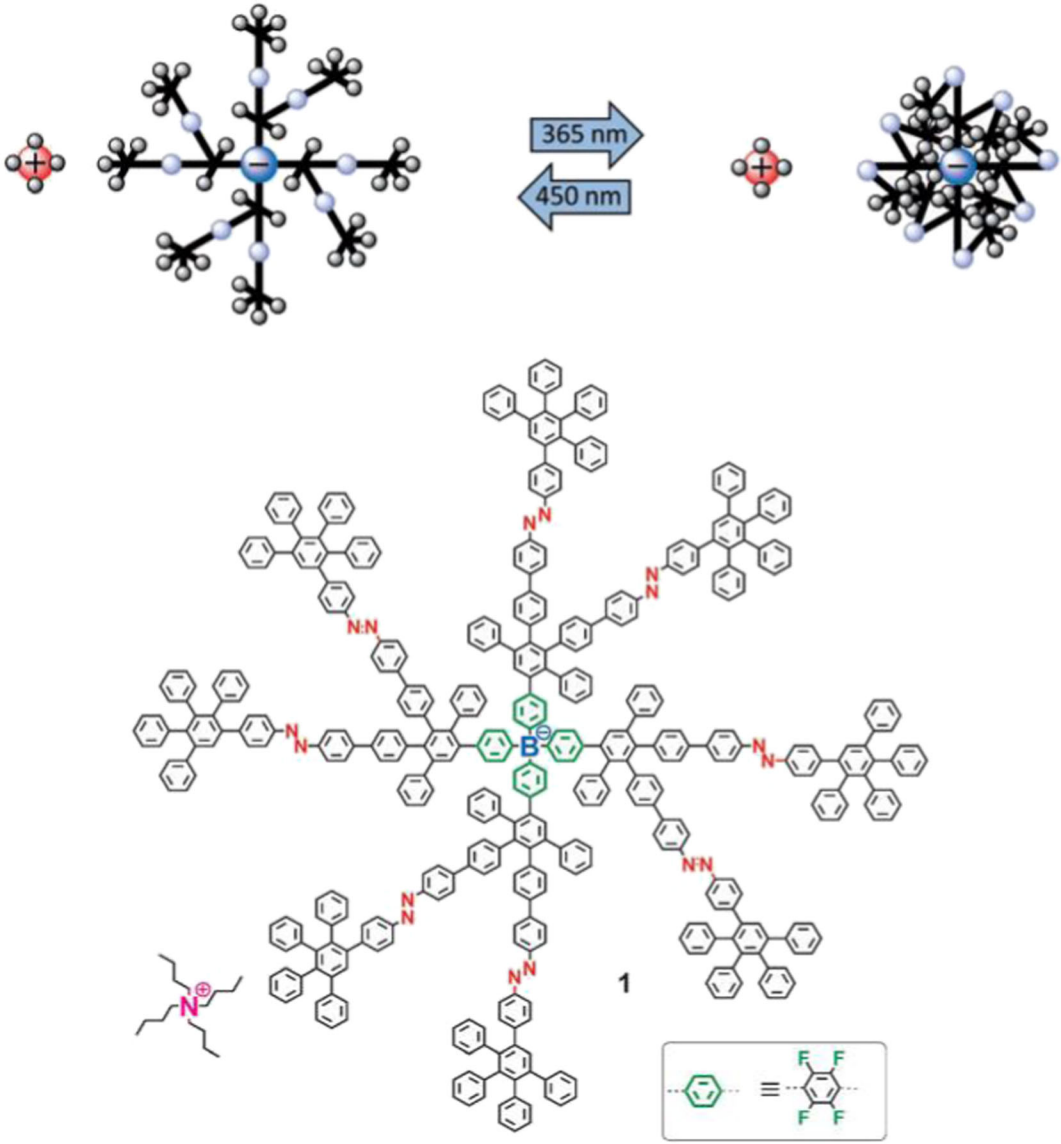
Illustration of size and density switching of a rigidly dendronized anion by azobenzene photoisomerization, and the structure of borate salt X, which bears eight azobenzene units throughout its dendrimer scaffold. (Reprinted with permission from John Wiley and Sons (2013)) (Nguyen et al. 2013a, b; Türp et al. 2011)

The application of such rigid WCIs was expanded to ion-pair shielding studies by introducing stimuli-responsive azobenzene groups into the dendrimer backbone (Fig. 4) (Nguyen et al. 2013a, b). Azobenzenes are known to undergo a reversible *cis/trans* photoisomerization upon exposure to 365 nm (*trans to cis*) and 450 nm (*cis to trans*) radiation. A second-generation dendrimer was synthesized from the boron-based anion where eight azobenzene moieties were placed between the first and second generations. The dendrimers were exposed to 450-nm light to promote the *cis* to *trans* isomerization to fully extend the backbone, and it was determined that these macromolecules had a hydrodynamic radius of ~ 1.9 nm. When these “open” PPDs were mixed with positively charged cations (tetrabutyl ammonium (TBA)), these positively charged species had less steric hindrance to the negatively charged core. However, upon irradiating the dendrimers with 365 nm to “close” the PPDs, the hydrodynamic radius shrunk to ~ 1.6 nm and the more densely packed dendrons shielded the anionic core from the TBA cations. The most significant effect on the size of these macromolecules was found in their conductivity, where the *cis* form had ~ 20–25% higher molar conductivity than the *trans* form, which also had a maximum value of 41.6 cm^2^ mol^−1^. This process opened to door to a new field of stimuli-responsive WCIs where the size (i.e., dendrimer generation) can be tuned to control the Coulombic interactions between ion pairs.

## Defining the Dendrimer scaffold

**Fig. 5:**
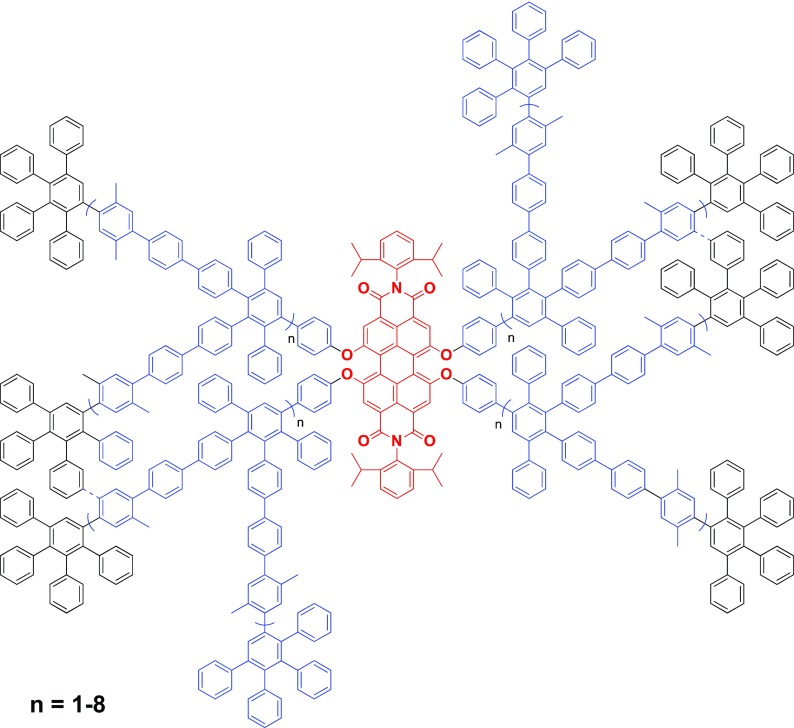
Polyphenylene dendrimer synthesized up to the ninth generation. (Reprinted with permission, copyright 2016, American Chemical Society) (Hammer and Müllen 2016)

One of the most significant achievements in the field of dendrimer synthesis occurred when polyphenylene dendrimers were synthesized up to the ninth-generation associated with a 1.9 MDa molecular weight, while maintaining their monodisperse (perylene diimide (PDI) less than 1.05) and shape-persistent nature, as characterized by transmission electron microscopy (TEM) and MALDI-TOF spectrometry (Nguyen et al. 2013a, b). It had previously been impossible to synthesize such large molecular weight, defined dendrimers due to steric limitations and unavoidable side reactions, especially for the surface dense PPD family. However, this obstacle was overcome by using cyclopentadienones with phenylene extensions to build the dendrimers that alleviated the steric strain of the synthesis, which has been previously shown to work for other dendrimer families (Ornelas et al. 2009; Petersen et al. 2015; Tomalia et al. 1987). As seen in Fig. 5, the PPDs were synthesized from a perylene diimide core up to the ninth-generation using these extended building blocks, and due to the quantitative and irreversible nature of the Diels-Alder cycloaddition, it was possible to obtain monodisperse samples of these dendrimers, as confirmed by MALDI-TOF (1.9 MDa g/mol). While other groups have been able to synthesize dendrimers up to such high generations, they were not able to achieve equivalent molecular weights and typically saw defects after the fourth or fifth generations (Ruiz et al. 2003). Furthermore, TEM was used to determine the size and shape-persistent nature of the PPDs, and these macromolecules showed a diameter of ~ 33 nm, which represents one of the largest reported dendrimers ever.

**Fig. 6:**
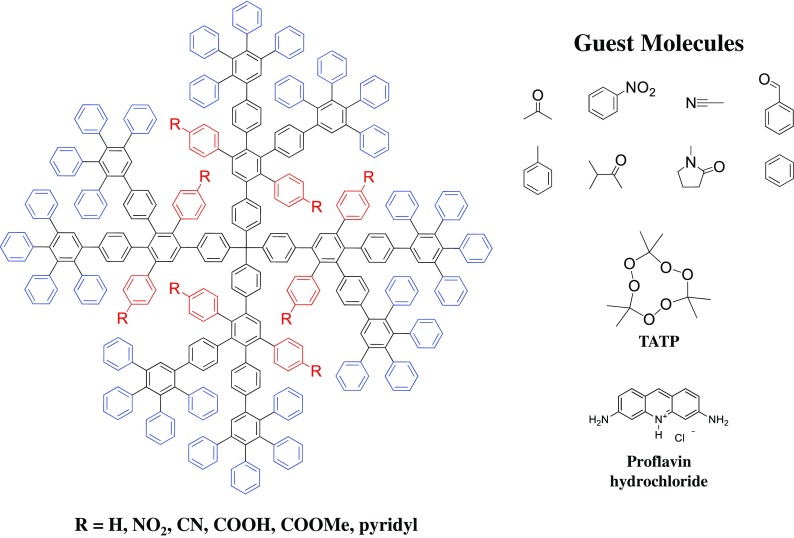
PPD scaffolds modified with polar or unpolar functionalities to tailor their encapsulation of guest species. (Reprinted with permission, copyright 2016, American Chemical Society) (Hammer and Müllen 2016)

It is critical to note the chemical functionalities within a dendrimer scaffold, as those species determine the hydrophobic or hydrophilic nature of the cavity, and, more importantly, the possibility to interact with guest molecules as a host system. Isothermal titration calorimetry (ITC) is an excellent tool to analyze the interactions between the host PPD and guest small molecules (Andreitchenko et al. 2008; Bauer et al. 2007; Chiad et al. 2013; Jansen et al. 1994; Köhn et al. 2001; Schlupp et al. 2001). Dendrimers that had unfunctionalized scaffolds (i.e., only had phenyl rings) were able to encapsulate unpolar small molecules (i.e., benzene, toluene, hexane), and ITC determined that this process was promoted through an entropically encouraged solvent exchange in solution. In contrast, when PPDs were chemically modified with polar scaffold groups (i.e., carboxylic acids, nitriles, nitro groups, esters) and exposed to polar molecules (acetonitrile, acetone, nitrobenzene, methanol), ITC identified that the binding between the dendrimer cavity and guest molecules was enthalpically driven through non-covalent forces (i.e., H-bridges, dipole interactions, π-πinteractions) (Fig. 6).

This concept was expanded to make PPDs nanocarriers for small molecules. A third-generation PPD was synthesized with 12 carboxylic acids in the scaffold and exposed to proflavin hydrochloride (Bauer et al. 2007). The intermolecular interactions between the cavity-based acid groups and the salt led the dendrimers to encapsulate 3–4 dye specifies per PPD, and these complexes even displayed solubility in unpolar solvents such as hexane and ether. This demonstrated a powerful ability to encapsulate polar species and act as a nanocarrier to transfer them through unpolar environments while maintaining the chemical structure.

While encapsulation and release studies are interesting, the significance of such experiments is real-world applications. With this in mind, attention shifted towards using these dendrimer nanocarriers as detectors for trace amounts of an explosive, triacetone peroxide (TATP) (Fig. 6), a highly dangerous material even in small quantities. Fourth-generation PPDs were synthesized with 56 pyridyl groups throughout their scaffolds to act as anchoring points for TATP guest molecules. These macromolecules were coated onto a quartz crystal microbalance (QCM) detector that was calibrated to account for residual materials from their synthesis (acetone, hydrogen peroxide, etc.), and a TATP-enriched nitrogen stream was flown over the sensors (Lubczyk et al. 2012). It was found that gaseous TATP molecules interacted with the pyridiyl groups within the dendrimer scaffold on the QCM detector, and a limit of detection as low as 0.1 ppm was observed. This is well within the limits of a viable chemosensor for detecting even trace amounts of the TATP explosive and made the encapsulation and release characteristics of PPDs particularly intriguing.

**Fig. 7:**
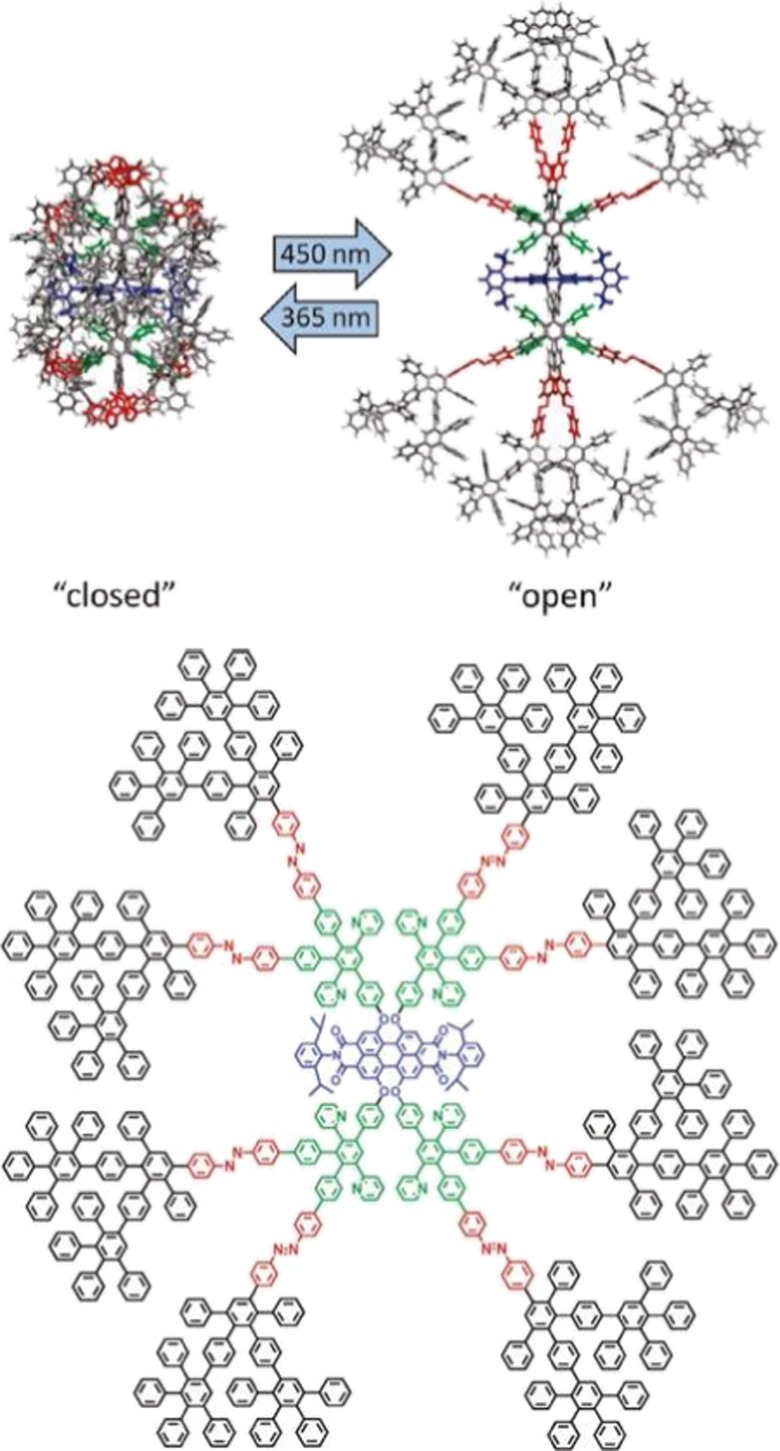
Photo-switchable PPDs for studies of stable encapsulation and release with small molecules. (Reprinted with permission, copyright 2012, American Chemical Society) (Nguyen et al. 2011)

While the encapsulation and release of small molecules within the scaffold of PPDs is important, the stability of the previously mentioned complexes is completely dependent on the intermolecular interactions between the host and guest molecules. Additionally, in applications such as therapeutic drug delivery, it is imperative to have a controlled release mechanism as to not unload the guest species prematurely. These are significant challenges to any dendrimer field, but especially for flexible dendrimers that can constantly undergo structural rearrangements based on their environment. When elastic dendrons expand and contract, it can limit intermolecular interactions or lead to the undesired leaching of the guest molecules. However, the rigid and shape-persistent nature of polyphenylene dendrimers can circumvent these obstacles if a controlled release mechanism of guest molecules out of the defined cavities were achieved. To this end, third-generation PPDs were synthesized with eight pyridyl binding sites throughout their scaffold and eight azobenzene functionalities between the first and second generation (Fig. 7) (Nguyen et al. 2011). As discussed in section “It all starts with the core,” azobenzene groups can undergo a reversible *cis*/*trans* photoisomerization upon exposure to electromagnetic radiation and it was this rearrangement between the open (*trans*) and closed forms (*cis*) that was an attractive approach to sterically trap guest molecules within the dendrimer cavities. Open PPDs were exposed to a solution of *p*-nitrophenol with the intent that it would interact with the interior pyridyl groups and then the solution was irradiated with 365-nm light to force the *trans*-*cis* isomerization. In an effort to test the stability of the complexes, they were purified via multiple precipitations and washings with methanol, and it was determined that the closed dendrimers were able to stably trap two small molecules per dendrimer. When the PPDs were exposed to 450-nm light to convert them back to the *trans*-isomer the p-nitrophenol guest molecules were released, which represents a controlled release mechanism that means PPDs have overcome the two major obstacles of encapsulation and release: 1() they do not undergo structural rearrangements due to their rigid backbone and (2) they have a controlled release mechanism to prevent premature leaching.

While the aforementioned stimuli-responsive PPDs demonstrated the ability to stably encapsulate two small molecules with a triggered release mechanism some applications require a higher encapsulation efficiency, alternative approaches to increase the number of guest species occurred. One such approach was to synthesize dendrimers with binding sites in their scaffold where guest molecules could be covalently connected to the PPDs through cleavable linkages. Second-generation PPDs were made with eight thiol groups throughout their interior to increase the scaffold’s polarity and act as binding sites for small molecules (Hammer et al. 2015; Hammer and Müllen 2016). These materials were exposed to thiol-functionalized nitrophenol derivatives and were bonded to the PPDs through oxidative disulfide bond formation with the interior thiols. Despite washing the conjugates with tetrahydrofuran (THF) multiple times (i.e., a good solvent for both the host and guest molecules) it was found that each dendrimer could bind up to four guest species, and the conjugates were stable to various solvents, even at elevated temperatures (~ 65 °C). Upon exposing the PPD conjugates to reductive conditions (dithiothreitol) the disulfide bonds were cleaved to release the guest nitrophenol groups. This process increased the loading capacity of the dendrimers as compared to the previous example, and the ability to reversibly attach small molecules within the scaffold of PPDs with a controlled release mechanism represents an appealing candidate for encapsulation and release applications.

## What is on the outside matters

**Fig. 8:**
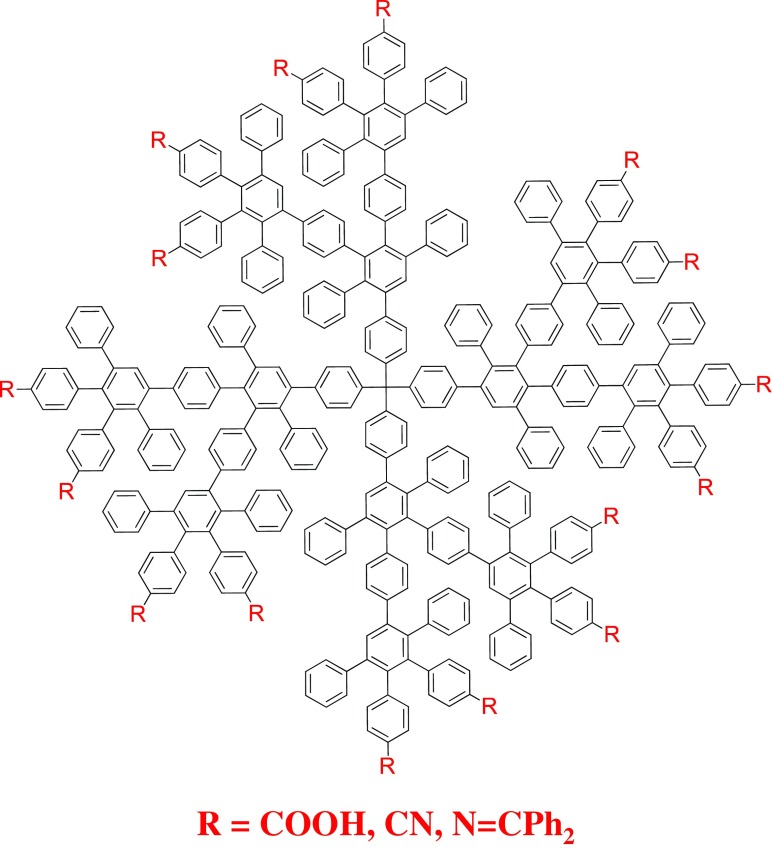
Second-generation PPD with polar surface functionalities. (Reprinted with permission, copyright 2016, American Chemical Society) (Hammer and Müllen 2016)

The surface functionalization of polyphenylene dendrimers influences their solubility and intermolecular interactions with other molecules (Dvornic 2006; Bauer et al. 2005; Fréchet and Hawker 1995; Gillies et al. 2004; Hammer and Müllen 2016). At the early stages of the PPD field, they were synthesized with mainly phenyl groups on the dendrimer exterior that led to modest solubility in hydrophobic solvents and limited intermolecular interactions (Wiesler et al. 2001). This was the result of a focus on using PPDs as nanographene precursors, which did not require the incorporation of heteroatoms at the time (Watson et al. 2001; Wu et al. 2003). However, as synthetic techniques and the imagination of chemists evolved, so too did the introduction of new functional groups onto the surface of PPDs. Early syntheses produced dendrimers with carboxylic acids, imines, nitriles, alcohols, or esters on their periphery, and a significant change in their solubilities and interactions with other molecules was seen (Fig. 8) (Dong et al. 2005; Lauter et al. 1998; Sun et al., 2013). Due to the limited solubility of unfunctionalized PPDs (i.e., only phenyl rings on the surface), they tend to aggregate into micron size particles through the interdigitation of their dendrons, especially in polar solvents (Baluschev et al. 2005; Clark et al. 2007; Grimsdale and Müllen 2001; Hussain et al. 2006). Yet, by adding polar groups to the dendrimer surface, a significant increase in their solubility was observed that gave rise to a decrease in their aggregation because of the repulsive forces between surface functionalities.

One of the first examples of chemically modifying the surface of PPDs and studying the results was when second-generation dendrimers were synthesized with 16 cyano-groups on their surface. These groups underwent an “a posteriori” basic hydrolysis to yield 16 carboxylic acids on the dendrimers. Single-molecule spectroscopy (SMS) looked at the interactions with these polar PPDs and a cyanine dye pinacyanol and it was determined that at low concentrations two dye groups could penetrate into the dendrimer scaffold but at higher concentrations they formed ion pairs with the carboxylic acids on the surface (Köhn et al. 2001; Zhang et al. 2000). Not only did this process demonstrate a concept to polarize PPDs but it also led to a concentration-based interaction with guest species to form either encapsulated hosts or dendrimers coated with the dye.

By changing the surface groups on PPDs, it is possible to manipulate their intermolecular interactions with guest molecules, and these variations can be applied for use as the active layer in QCM detectors as a method to detect a wide range of volatile organic compounds (VOCs) (Bachar et al. 2013; Bayn et al. 2013; Schlupp et al. 2001). Devices were fabricated where the active layer was composed of second-generation dendrimers that had surfaces modified with either carboxylic acids, nitriles, or diphenylmethyleneamines. These detectors were exposed to gaseous molecules (i.e., acetophenone, aniline, benzonitrile) and, using positron emission tomography (PET), it was determined that each active layer could uptake ~ 5 × 10^15^ of the gaseous, polar molecules. This study represented an impressive advancement relating how synthetic chemical modifications of dendrimers can impact their intermolecular interactions with guest molecules and how those interactions can influence the location of small molecules (i.e., either with the dendrimer scaffold or around their exterior).

In addition to tuning the solubilities of PPDs through surface modifications, it is also possible to manipulate their assembly properties in the active layer of a device. For example, PPDs were synthesized with dithiolane or thiomethyl groups on their surface and mixed with gold nanoparticles to form nanocomposites, which were used as a selective layer in a chemoresistor sensor for VOCs (John and Tour 1997; Lee et al. 2003; Tan et al. 2015; Taubert et al. 2003; Zhang et al. 2014a, b). The dendrimer stabilized nanocomposites led to an increased detection of organic solvents such as benzene, toluene, and trichlorobenzene. Alternatively, dithiolane functionalized PPDs were synthesized from a four-armed perylene diimide (PDI) core and used as a ligand spacer between a silver plate and sphere. This nanoscopic assembly of the dendrimers led to plasmonic gap resonance of the PDI core due to the molecularly thick layer between the plane and sphere, and this led to the quenching of its fluorescence onto the silver plate yet a fluorescence amplification of ~ 1000 times on the silver sphere through plasmonic resonators (Hussain et al. 2006; Liu et al. 2003; Taubert et al. 2003).

**Fig. 9:**
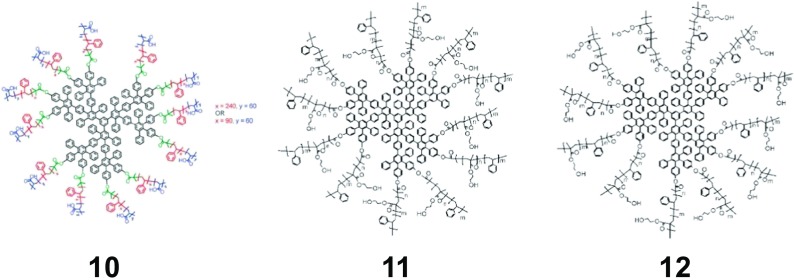
Polymer functionalized PPDs. (Reprinted with permission, copyright 2016, American Chemical Society) (Hammer and Müllen 2016)

It is also possible to surface-functionalize polyphenylene dendrimers with polymers to even further control their properties. Dendrimers were synthesized and using an a posteriori grafting techniques, atom transfer radical polymerization (ATRP) initiators have been placed on their surface. Poly(2-hydroxylethyl methacrylate) (PHEMA)-b-poly (styrene) (PS) (Fig. 9 molecule 12) and PS-b-PHEMA (Fig. 9 molecule 11) diblock copolymers were grown from the PPD exterior to achieve stimuli-responsive polymer shells around the dendrimers (i.e., undergo conformational rearrangements depending on their environment) (Hammer and Müllen 2016; Yin et al. 2007) When the macromolecules were dissolved in a good solvent for both blocks, the hydrodynamic radius of the dendrimers was ~ 15.6 nm, but when exposed to a poor solvent for on block (5 vol.% methanol in THF for PS), the radius collapsed to ~ 8.5 nm. By introducing amphiphilic diblock copolymers to the dendrimer surface, it was possible to expand their solubility, while also incorporating a stimuli-responsive exterior coating for controlling the size and surface function of the PPDs.

Another “multicore shell” dendrimer design involved growing PS-b-PAA diblock copolymers (Fig. 9 molecule 10) from the surface of PPDs (Yin et al. 2007). The acrylic acids of the dendrimers were used to coordinate titanium dioxide (TiO_2_) nanoparticles resulting in polymer composites, and the hydrophobic PPD and PS components were removed by sequential hydrolysis, condensation and calcination to achieve hollow nanocomposites (Zhi et al. 2007). By changing the dendrimer size, PS block length was possible to control the open volume of the hallow nanocavities, while the PAA layer was utilized to change the template size. This process efficiently achieved hollow metal oxide composites with tuneable dimensions that have shown promise in catalysis, VOCs sensing, and lithium ion battery applications.

**Fig. 10:**
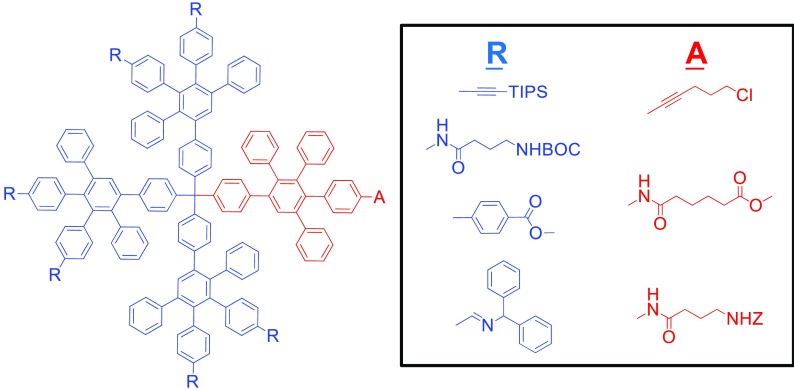
PPDs asymmetrically modified with different polar groups. (Reprinted with permission, copyright 2016, American Chemical Society) (Hammer and Müllen 2016)

A very significant challenge to all fields of dendrimer chemistry is the synthesis of well-defined, asymmetric macromolecules with controlled chemical properties (Fig. 10) (Gillies and Fréchet 2005). Yet, the synthetic versatility of PPDs, and their cyclopentadienone building blocks, has made it possible to achieve such complex structures. Four-arm, asymmetric PPDs have been synthesized with one arm possessing an anchoring group such as an amine, carboxylic acid, amide, or alkyl chloride that was utilized to attach the dendrimers to surfaces and act as binding sites for proteins (Walther and Müller 2013; Yang et al. 2008). Another approach led to the synthesis of a first-generation PPD with a single biotin unit on one dendron while the other three arms possessed perylenemonoimide (PMI) groups. Upon mixing these asymmetric macromolecules with Tween 20 detergent, the biotin anchoring site interacted with the surfactant to form dendritic complexes. Furthermore, these dendrimers were observed to bind the protein streptavidin at the biotin anchor site, which resulted in the PMI units acting as a fluorescent tag for the protein (Minard-Basquin et al. 2003).

**Fig. 11:**
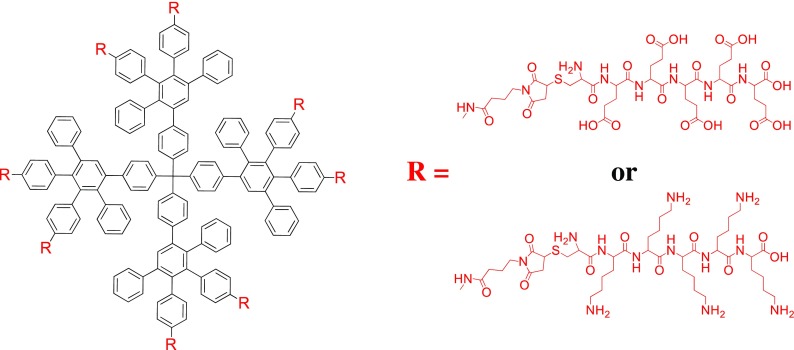
Synthesis of poly (L-lysine) from the surface of PPDs (Reprinted with permission from John Wiley and Sons (2015)) (Dong et al. 2005; Koynov et al. 2007; Mondeshki et al. 2006)

Recently, there has been a strong focus on using dendrimers in biological applications such as cell uptake or gene therapies and as nanocarriers for therapeutic drugs, because of the ability to synthesize chemically defined and monodisperse materials (Kannan et al. 2014). For polyphenylene dendrimers, one of the first approaches involved first- and second-generation PPDs functionalized with surface-bound amine groups that were used to couple the C-terminus-activated carboxylic acid group of poly (L-lysine) (Fig. 11) (Dong et al. 2005; Koynov et al. 2007; Mondeshki et al. 2006). These dendrimer-amino acid complexes were water-soluble, and the length of the peptide chain influenced the conformation of the assembly. When a small to medium or large peptides was attached to PPDs they formed α-helical or β-sheet structures, respectively.

Alternatively, dendrimers modified with ATRP surface initiators were employed to polymerize 2-tertbutoxycarbonylaminoethyl methacrylate, and after removing the t-boc groups, the resulting surface amines could stably complex with DNA and plasmid DNA, even at low concentrations (Dong et al. 2005; Koynov et al. 2007; Mondeshki et al. 2006). Furthermore, when these dendrimers were built around a PDI core, they could be used for in vivo studies as a fluorescent tag, and they were applied to stain the extracellular matrix (EDM) in animal tissues at physiological PHs. When the surface-bound amine groups were quanternized to their ammonium cations they could even transport through cell membranes. Additional experiments revealed that these PPDs complexed with RNA sequences that target the mid-gut chitinase gene (CHT10-dsRNA) in Asian corn borer and could suppress the developmental gene expression (i.e., prevents growth). These dendrimer/RNA assemblies were orally fed to freshly hatched larvae, which stunted their growth, and this is one of the first reported non-viral gene therapies that demonstrated the ability to control gene expression.

**Fig. 12:**
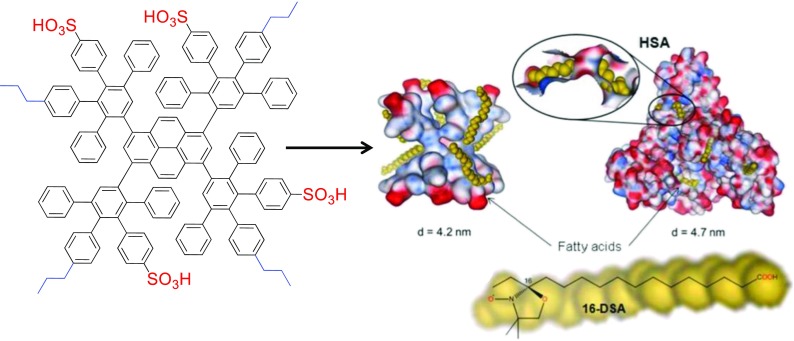
PPDs with a “patched” surface functionalization that were used for cell uptake and drug complexation experiments (Reprinted with permission from John Wiley and Sons (2014)). (Stangenberg et al. 2014a, b, Stangenberg et al. 2015)

Many synthetic molecules that are successful in biological applications are actually modeled to mimic naturally occurring compounds such as therapeutic drugs, proteins, and fatty acids. Thus, a tremendous accomplishment in the field of polyphenylene dendrimers was the synthesis of “patched” surface dendrimers that had equal quantities of nonpolar (propyl chain) and polar (sulfonic acid) groups. (Fig. 12) (Stangenberg et al. 2014a, b, Stangenberg et al. 2015). Ideally, the propyl chains were intended to interact with nonpolar species (fats, oils, etc.) and the sulfonic acids increasing the water solubility and intermolecular interactions with polar molecules, with the shape-persistent nature of PPDs preventing any conformational rearrangements of those groups based on their environment. A clever detail of this design was the minute distances between the propyl and sulfonic acid groups on the dendrimer surface, because this led to local areas of contrasting polar and unpolar regimes, which produced frustrated solvent structures. Here, the PPDs were soluble in water and polar organic solvents (THF, acetone, methanol) and experiments determined that the solvent molecules were actually trapped at the different regimes around the dendrimer instead of fully solvating the macromolecules. Experiments were carried out with first through third generations of these patched surface dendrimers to bind 16-DOXYL-stearic acid, a spin-labeled fatty acid, and complex doxorubicin (anti-cancer therapeutic drug), as compared to human serum albumin (HSA), a naturally occurring HSA protein. Studies showed that the PPD could bind nine 16-DOXYL-stearic acids while the HSA could only bind seven, and both macromolecules could encapsulate one doxorubicin molecule and successfully transferred it into human lung carcinoma cells. It should be noted that cell lysis and fluorimetry revealed a much higher cell uptake for PPDs versus HSA. Furthermore, cell viability studies were carried out using zebrafish embryos (6–72 h post-fertilization) and the PPD and HSA demonstrated minimal cell toxicity, while a positively charged poly (amidoamine) (PAMAM) reference dendrimer displayed significantly higher toxicities. An even more significant accomplishment of these patched surface dendrimers was that they showed the ability to cross the ever-challenging blood-brain barrier (BBB) and even penetrate the tightly packed brain endothelial cells at up to a 37% efficiency.

While these results were very encouraging, it was still necessary to understand the fundamental forces behind the high cell uptake and low toxicity of patched surface dendrimers, so a series of experiments were designed to observe the influence and the type and relative ratios of the polar and nonpolar groups. First- and second-generation dendrimers were synthesized with sulfonic, carboxylic, or phosphonic acids as the polar group, and n-propyl, butyl, hexyl, or isopropyl chains as the nonpolar functionality to observe the influence of the type of chemical modification (Hammer et al. 2017). Additionally, PPDs were synthesized with either a 1:1 or 2:1 ratio of polar/nonpolar groups (i.e., two sulfonic acids per one propyl chain) to investigate how the relative ratio influences their properties. Results confirmed that the size (generation) and type of polar or nonpolar functionality did not affect the high cell uptake into human lung carcinoma cells or low cytotoxicity (< 10%) of the dendrimers. However, a significant development determined that increasing the ratio of polar/nonpolar groups from 1:1 to 2:1 M equivalents resulted in a severe decrease in cell uptake and increased cell toxicity of the dendrimers since they were observed to aggregate on the cell membranes, as confirmed by confocal microscopy. This suggested that the chemical modification of patched surface PPDs with various polar and nonpolar groups did not influence their interactions with cells, but it was the delicate 1:1 M balance between these species that forms an optimal lipophilic shell around the dendrimers to promote efficient cell uptake with minimal toxicity. The understanding that it is not solely the chemical modifications of patched surface dendrimers that influences their properties, but also the lipophilic balance of their exterior is a major achievement, and provides an understanding how to rationally design molecular biomimics in the future.

## 3-D polyphenylene dendrimers to functional 2-D nanographenes

**Fig. 13:**
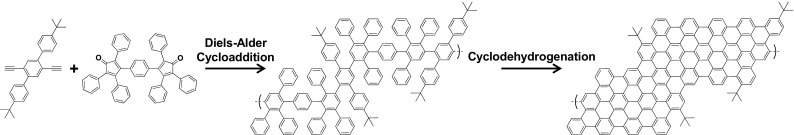
Introducing polyphenylenes as precursors for graphene nanoribbons

Dendrimers are considered monodisperse, 3-D globular structures, and many dendrimer structures are restricted to these morphologies (Newkome and Shreiner 2008). But one of the most impressive abilities of polyphenylene dendrimers is their ability to be converted from 3-D globular macromolecules to 2-D nanographene derivatives, as this unique process has led to the synthesis of molecularly defined graphenes with tremendous electronic and mechanical properties (Türp et al. 2012; Watson et al. 2001) Such characteristics of graphenic materials are dependent on its uniformity, both geometrically and chemically, any integration of heteroatoms (i.e., nitrogen, oxygen, phosphorus, boron, halides), aspect ratio that can determine its band gap (i.e., length to width ratio of the molecule), and the edge structure (Narita et al. 2015). While many “top down” approaches produce large amounts of graphene derivatives, these products typically lack definition in most of the aforementioned areas that dictate properties, so attention has shifted towards more “bottom up” synthetic strategies because they afford control over the size and chemical modification of the graphene species. One of the first reported syntheses of nanographenes occurred when polyphenylene dendrimers were synthesized through repetitive Diels-Alder cycloaddition reactions as illustrated in Fig. 13 (Wu et al. 2003). After the dendritic ribbon was formed, it was exposed to FeCl_3_ to induce a cyclodehydrogenation of the ribbon into a 2-D graphene nanoribbon, which represented one of the first examples of synthesizing a well-defined graphene derivative (Bieri et al. 2009; Müller and Müllen 2007; Narita et al. 2015; Andreitchenko et al. 2008; Wu et al. 2003). While limited solubility stunted the ability to achieve large or chemical modifications at the time, this process ignited the imagination of synthetic to pursue more complex graphenic structures. Thus, PPDs were the first reported graphene precursors where their synthetic details (i.e., geometry and size) could be used to tailor the graphene structures, and, more importantly, the properties of those molecules (Angelova et al. 2013; Dössel et al. 2011; El Hamaoui et al. 2007; Müller and Müllen 2007; Narita et al. 2013, 2015; Rao et al. 2014; Schlütter et al. 2014; Wu et al. 2005)

Recent advances in the field of PPDs as precursors for synthetic graphene nanoribbons (GNRs) have involved attaching solubilizing chains on the edges of the macromolecules to improve solution processing, controlling the edge structure, and introducing heteroatoms into the system.

**Fig. 14:**
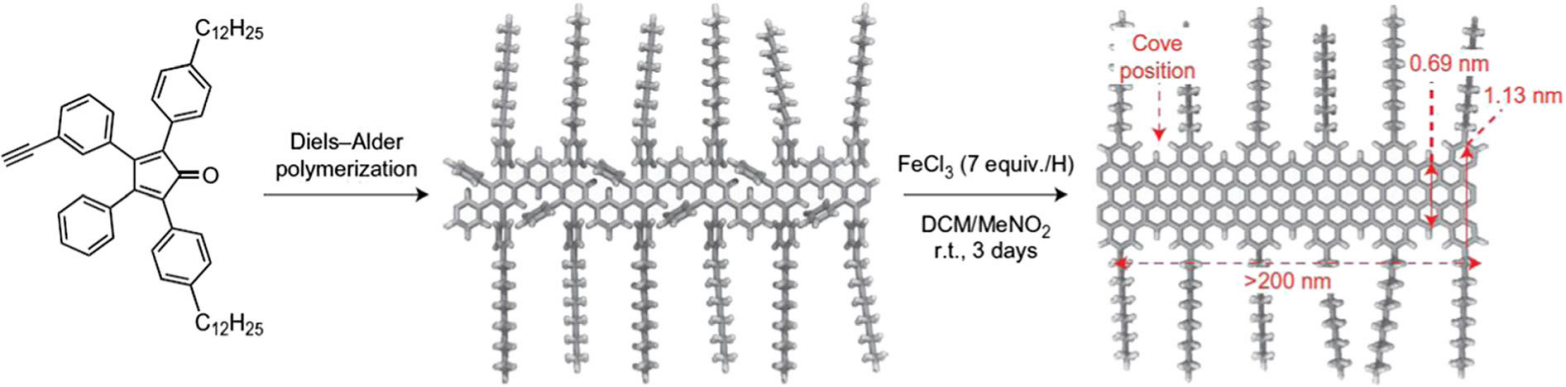
Processable graphene nanoribbons from polyphenylene precursors (Reprinted with permission from Nature Publishing Group (2014)). (Narita et al. 2015)

Figure 14 illustrates a sequence of reactions that converted a linear PPD into one of the largest GNRs recorded with widths ~ 1.0 nm and lengths up to 600 nm. These materials displayed more promising charge carrier mobilities and electronic properties as compared to their semiconducting polymer counterparts, and they had a band gap of ~ 1.88 eV. This is significant as graphene does not inherently have a band gap so it is necessary to utilize geometric confinement in the molecule to induce a band gap, which is required for the electronic applications due to the necessity to turn the devices on and off. Previously, our group has been able to synthesize GNRs with band gaps between 1.2 and 3.6 eV, which represents the ability to determine their electronic properties based on synthetic design (Narita et al. 2013, 2015). A new era of graphene synthesis was established when we accessed GNR no longer by classical polymer synthesis in solution but rather by the polymerization of suitable monomers after their immobilization on surfaces. This offered the additional advantage of monitoring the GNR formation by scanning tunneling microscopy. The ability to synthetically control the dimensions, uniformity, and electronic band structure of GNRs has opened the door to the next generation of graphene-based semiconductors. Furthermore, GNRs, depending on the nature of their periphery, can possess edge localized states whose associated spins offer an entry into spintronics (Ruffieux et al. 2016). The limited stability of GNRs with the required zigzag edges made another approach particularly valuable: there we synthesized GNRs, again via repetitive Diels-Alder polymerization and subsequent cyclodehydrogenation of the twisted precursor polyphenylenes, but at the same time attached stable radicals at the peripheries. These radical centers would then inject their spin density into the semiconducting GNRs (Slota et al. 2018), thus furnishing stable GNRs with spins at their edges.

## Looking forward

Polyphenylene dendrimers represent a special class of macromolecules because of their combination of structural perfection, shape-persistence, stability, and versatile syntheses. The initial stages of PPD chemistry were mainly used to transform 3-D structures into molecularly defined nanographenes, and this has stimulated the field of synthetic graphene to this day. Yet, as synthetic processes have expanded, it has made even more complicated structures possible and this has led to applications ranging from organic electronics to molecular biomimics. The synthetic versatility and stability of PPDs is what allows their targeted chemical functionalization with a tremendous number of chemical species (i.e., hydrophobic or hydrophilic groups, polymers, peptides, binding agents), and through this synthetic ingenuity new applications have been realized. PPDs have been used as the active layer in organic electronics, VOC detectors for their ability to make uniform and responsive layers; weakly coordinating ions for their capacity to shield interior ions with their shape-persistent dendrons, hosts for the encapsulation of guest molecules with triggered release mechanisms, and even as nanocarriers to transport therapeutic drugs across cell membranes. These accomplishments are a result of synthetic advancements combined with the versatility to functionalize PPDs at all three levels (i.e., core, scaffold, and surface) to tailor their properties.

It is now necessary to shift focus towards the future and complementing innovative synthetic strategies with their controlled assembly in route to the next generation of material’s applications. It has already been shown that PPDs have high cell uptake and low toxicity, and transport therapeutic drugs through cell membranes. Yet, this is not enough for clinical trials because it is necessary to improve the loading capacity of such nanocarriers while introducing more efficient release mechanisms to fabricate even better cancer treatments. Furthermore, most macromolecule-based cancer therapeutics rely on the enhanced permeation and retention (EPR) effect, but this is statistically insufficient at effectively finding and penetrating cancer cells, which encourages chemically modifying dendrimers with targeting agents to significantly improve their selective uptake into cancer cells.

Dendrimers represent a fantastic field of chemistry where synthesis and application can bond towards achieving solutions most other fields cannot, but it is in pursuing the next and more challenging obstacles that creativity and understanding must be introduced. It is time to move past comfortable experiments with well-documented materials in hopes of stumbling across a new application and instead strive to realize the next synthetic breakthrough towards macromolecules that were previously unimaginable and which also have the desired assembly behavior encoded.

## References

[CR1] Andreitchenko EV, Bauer RE, Kreutz C, Baumgarten M, Bargon J, Müllen K (2008). Size and shape variation of polyphenylene dendrimers through the heterogeneous hydrogenation of embedded triple bonds. Macromolecules.

[CR2] Angelova P, Vieker H, Weber NE, Matei D, Reimer O, Meier I, Kurasch S, Biskupek J, Lorbach D, Wunderlich K, Chen L, Terfort A, Klapper M, Müllen K, Kaiser U, Gölzhäuser A, Turchanin A (2013). A universal scheme to convert aromatic molecular monolayers into functional carbon nanomembranes. ACS Nano.

[CR3] Astruc Didier, Chardac Françoise (2001). Dendritic Catalysts and Dendrimers in Catalysis. Chemical Reviews.

[CR4] Bachar N, Liberman L, Muallem F, Feng X, Müllen K, Haick H (2013). Sensor arrays based on polycyclic aromatic hydrocarbons: chemiresistors versus quartz-crystal microbalance. ACS Appl Mater Interfaces.

[CR5] Baluschev S, Jacob J, Avlasevich YS, Keivanidis PE, Miteva T, Yasuda A, Nelles G, Grimsdale AC, Müllen K, Wegner G (2005). Enhanced operational stability of the up-conversion fluorescence in films of palladium-porphyrin end-capped poly (pentaphenylene). ChemPhysChem.

[CR6] Bauer Roland E., Grimsdale Andrew C., Müllen Klaus (2005). Functionalised Polyphenylene Dendrimers and Their Applications. Topics in Current Chemistry.

[CR7] Bauer, R. E., Clark, C. G., & Muïlen, K. (2007). Precision host–guest chemistry of polyphenylene dendrimers. 10.1039/b617666f

[CR8] Baumgarten, M. (2015). “Dendrimers” in: Ullmann’s encyclopedia of industrial chemistry. 10.1002/14356007.s08_s01, Dendrimers

[CR9] Bayn Alona, Feng Xinliang, Müllen Klaus, Haick Hossam (2013). Field Effect Transistors Based on Polycyclic Aromatic Hydrocarbons for the Detection and Classification of Volatile Organic Compounds. ACS Applied Materials & Interfaces.

[CR10] Bernhardt S, Baumgarten M, Wagner M, Müllen K (2005). Multiple functionalization of benzophenones inside polyphenylene dendrimers—toward entrapped ions and radicals. J Am Chem Soc.

[CR11] Bernhardt S, Kastler M, Enkelmann V, Baumgarten M, Müllen K (2006). Pyrene as chromophore and electrophore: encapsulation in a rigid polyphenylene shell. Chem Eur J.

[CR12] Bieri M, Treier M, Cai J, Aït-Mansour K, Ruffieux P, Gröning O, Gröning P, Kastler M, Rieger R, Feng X, Müllen K, Fasel R (2009) Porous graphenes: two-dimensional polymer synthesis with atomic precision. Chem Commun:6919. 10.1039/b915190g 10.1039/b915190g 19904347

[CR13] Bieri Marco, Nguyen Manh-Thuong, Gröning Oliver, Cai Jinming, Treier Matthias, Aït-Mansour Kamel, Ruffieux Pascal, Pignedoli Carlo A., Passerone Daniele, Kastler Marcel, Müllen Klaus, Fasel Roman (2010). Two-Dimensional Polymer Formation on Surfaces: Insight into the Roles of Precursor Mobility and Reactivity. Journal of the American Chemical Society.

[CR14] Blankenburg S, Cai J, Ruffieux P, Jaafar R, Passerone D, Feng X, Müllen K, Fasel R, Pignedoli CA (2012). Intraribbon heterojunction formation in ultranarrow graphene nanoribbons. ACS Nano.

[CR15] Brocorens P, Lazzaroni R, Bredas JL (2007). Molecular modeling simulations of the morphology of polyphenylene dendrimers. J Phys Chem B.

[CR16] CAMINADE A, LAURENT R, MAJORAL J (2005). Characterization of dendrimers. Advanced Drug Delivery Reviews.

[CR17] Carbone P, Negri F, Müller-Plathe F (2007). A coarse-grained model for polyphenylene dendrimers: switching and backfolding of planar three-fold core dendrimers. Macromolecules.

[CR18] Chiad K, Grill M, Baumgarten M, Klapper M, Müllen K (2013). Guest uptake by rigid polyphenylene dendrimers acting as a unique dendritic box in solution proven by isothermal calorimetry. Macromolecules.

[CR19] Clark, C. G., Wenzel, R. J., Andreitchenko, E. V, Steffen, W., Zenobi, R., & Muïlen, K. (2007). Solvophobically-driven 3-D self-assembly of exploded”-type polyphenylene dendrimers. 10.1039/b617667b, 31, 1300

[CR20] Dong HK, Hernandez-Lopez JL, Liu J, Mihov G, Zhi L, Bauer RE (2005). Multilayer films fabricated from oppositely charged polyphenylene dendrimers by electrostatic layer-by-layer assembly. Macromol Chem Phys.

[CR21] Dössel L, Gherghel L, Feng X, Müllen K (2011). Graphene nanoribbons by chemists: nanometer-sized, soluble, and defect-free. Angewandte Chemie - Int Edition..

[CR22] Dvornic PR (2006). PAMAMOS: The First Commercial Silicon-Containing dendrimers and their applications. J Polymer Sci.

[CR23] El Hamaoui B, Zhi L, Wu J, Li J, Lucas NT, Tomović Ž (2007). Solid-state pyrolysis of polyphenylene-metal complexes: a facile approach toward carbon nanoparticles. Adv Funct Mater.

[CR24] Feng X, Liang Y, Zhi L, Thomas A, Wu D, Lieberwirth I, Kolb U, Müllen K (2009). Synthesis of microporous carbon nanofibers and nanotubes from conjugated polymer network and evaluation in electrochemical capacitor. Adv Funct Mater.

[CR25] Tomalia Donald A., Fréchet Jean M. J. (2002). Discovery of dendrimers and dendritic polymers: A brief historical perspective*. Journal of Polymer Science Part A: Polymer Chemistry.

[CR26] Fréchet JMJ, Hawker CJ (1995). Hyperbranched polyphenylene and hyperbranched polyesters: new soluble, three-dimensional, reactive polymers. React Funct Polym.

[CR27] Gillies ER, Fréchet JMJ (2005). Dendrimers and dendritic polymers in drug delivery. Drug Discov Today.

[CR28] Gillies ER, Jonsson TB, Fréchet JMJ (2004). Stimuli-responsive supramolecular assemblies of linear-dendritic copolymers. J Am Chem Soc.

[CR29] Grayson Scott M., Fréchet Jean M. J. (2001). Convergent Dendrons and Dendrimers:  from Synthesis to Applications. Chemical Reviews.

[CR30] Grimes KD, Gupte A, Aldrich CC (2010). Copper (II)-catalyzed conversion of aryl/heteroaryl boronic acids, boronates, and trifluoroborates into the corresponding azides: substrate scope and limitations. Synthesis.

[CR31] Grimsdale AC, Müllen K (2001). 1-, 2-, and 3-dimensional polyphenylenes—from molecular wires to functionalised nanoparticles. Chemical Record Chem Rec.

[CR32] Grimsdale AC, Bauer R, Weil T, Tchebotareva N, Wu J, Watson M, Müllen K (2002). The chemical desymmetrisation of two-and three-dimensional polyphenylenes as a key step to functional nanoparticles. Synthesis.

[CR33] Gronheid R, Hofkens J, Köhn F, Weil T, Reuther E, Müllen K, De Schryver FC (2002). Intramolecular Förster energy transfer in a dendritic system at the single molecule level. J Am Chem Soc.

[CR34] Haberecht MC, Schnorr JM, Andreitchenko EV, Clark CG, Wagner M, Müllen K (2008). Tris(2,2′-bipyridyl) ruthenium (II) with branched polyphenylene shells: a family of charged shape-persistent nanoparticles. Angewandte Chemie Int Edition.

[CR35] Hammer BAG, Müllen K (2016). Dimensional evolution of polyphenylenes: expanding in all directions. Chem Rev.

[CR36] Hammer BAG, Moritz R, Stangenberg R, Baumgarten M, Müllen K (2015). The polar side of polyphenylene dendrimers. Chem Soc Rev.

[CR37] Hammer BAG, Wu Y, Fischer S, Liu W, Weil T, Müllen K (2017). Controlling cellular uptake and toxicity of polyphenylene dendrimers by chemical functionalization. Chembiochem.

[CR38] Hernandez-Lopez, J. L., Bauer, R. E., Chang, W.-S., Glasser, G., Grebel-Koehler, D., Klapper, M., … Knoll, W. (2003). Functional polymers as nanoscopic building blocks

[CR39] Herrmann, A., Weil, T., Sinigersky, V., Wiesler, U.-M., Vosch, T., Hofkens, J., … Müllen, K. (2001). Polyphenylene dendrimers with perylene diimide as a luminescent core 10.1002/1521-3765(20011119)7:22<4844::aid-chem4844>3.0.co;2-1 11763453

[CR40] Hussain I, Wang Z, Cooper AI, Brust M (2006). Formation of spherical nanostructures by the controlled aggregation of gold colloids. Langmuir.

[CR41] Imai M, Arai T (2002). Synthesis and photochemical properties of polyphenylene dendrimers with photoreactive stilbene core. Tetrahedron Lett.

[CR42] Jansen, J.F.G.A., De Brabander-Van Den Berg, E.M.M., & Meijer, E.W. (1994). Encapsulation of guest molecules into a dendritic box 10.1126/science.266.5188.1226 17810265

[CR43] John JA, Tour JM (1997). Synthesis of polyphenylene derivatives by thermolysis of enediynes and dialkynylaromatic monomers. Tetrahedron.

[CR44] Kannan R. M., Nance E., Kannan S., Tomalia D. A. (2014). Emerging concepts in dendrimer-based nanomedicine: from design principles to clinical applications. Journal of Internal Medicine.

[CR45] Kimura M, Sakaguchi A, Ohta K, Hanabusa K, Shirai H, Kobayashi N (2003). Selective ligation to sterically isolated metallophthalocyanines. Inorg Chem.

[CR46] Köhn, F., Hofkens, J., Wiesler, U.-M., Cotlet, M., Van Der Auweraer, M., Müllen, K., & De Schryver, F. C. (2001). Single-molecule spectroscopy of a dendrimer-based host ± guest system. 10.1002/1521-3765(20011001)7:19<4126::AID-CHEM4126>3.0.CO;2-M 10.1002/1521-3765(20011001)7:19<4126::aid-chem4126>3.0.co;2-m 11686590

[CR47] Koynov K, Mihov G, Mondeshki M, Moon C, Spiess HW, Müllen K, Butt HJ, Floudas G (2007). Diffusion and conformation of peptide-functionalized polyphenylene dendrimers studied by fluorescence correlation and 13C NMR spectroscopy. Biomacromolecules.

[CR48] Lauter U, Meyer WH, Enkelmann V, & Wegner G. (1998). Supramolecular structures of poly (p-phenylenes) with oxyethylene side chains and their mixtures with lithium salts. *Macromol.* Chem Phys, 1992, 29–2. 10.1002/(SICI)1521-3935(19981001)199:10<2129::AID-MACP2129>3.0.CO;2-S

[CR49] Lee JO, Lientschnig G, Wiertz FGH, Struijk M, Janssen RAJ, Egberink R, Dekker C (2003). Electrical transport study of phenylene-based π-conjugated molecules in a three-terminal geometry. In Annals New York Academy Sci.

[CR50] Li C, Liu M, Pschirer NG, Baumgarten M, Müllen K (2010). Polyphenylene-based materials for organic photovoltaics. Chem Rev.

[CR51] Liu D, De Feyter S, Cotlet M, Stefan A, Wiesler UM, Herrmann A (2003). Fluorescence and intramolecular energy transfer in polyphenylene dendrimers. Macromolecules.

[CR52] Lubczyk D, Grill M, Baumgarten M, Waldvogel SR, Müllen K (2012). Scaffold-optimized dendrimers for the detection of the triacetone triperoxide explosive using quartz crystal microbalances. ChemPlusChem.

[CR53] Maraval Valérie, Pyzowski Jaroslaw, Caminade Anne-Marie, Majoral Jean-Pierre (2003). “Lego” Chemistry for the Straightforward Synthesis of Dendrimers. The Journal of Organic Chemistry.

[CR54] Melnikov SM, Yeow EKL, Uji-i H, Cotlet M, Müllen K, De Schryver FC, Hofkens J (2007). Origin of simultaneous donor-acceptor emission in single molecules of peryleneimide-terrylenediimide labeled polyphenylene dendrimers. J Phys Chem B.

[CR55] Métivier R, Kulzer F, Weil T, Müllen K, Basché T (2004). Energy transfer rates and pathways of single donor chromophores in a multichromophoric dendrimer built around a central acceptor core. J Am Chem Soc.

[CR56] Mihov G, Grebel-Koehler D, Lübbert A, Vandermeulen GWM, Herrmann A, Klok HA, Müllen K (2005). Polyphenylene dendrimers as scaffolds for shape-persistent multiple peptide conjugates. Bioconjug Chem.

[CR57] Minard-Basquin C, Weil T, Hohner A, Rädler JO, Müllen K (2003). A polyphenylene dendrimer-detergent complex as a highly fluorescent probe for bioassays. J Am Chem Soc.

[CR58] Mondeshki M, Mihov G, Graf R, Spiess HW, Müllen K, Papadopoulos P, Gitsas A, Floudas G (2006). Self-assembly and molecular dynamics of peptide-functionalized polyphenylene dendrimers. Macromolecules.

[CR59] Morgenroth F (1998). Spherical polyphenylene dendrimers via Diels–Alder reactions: the first example of an A_4_B building block in dendrimer chemistry. Chem. Commun.

[CR60] Morgenroth F, Müllen K (1997). Dendritic and hyperbranched polyphenylenes via a simple Diels-Alder route. Tetrahedron.

[CR61] Morgenroth F, Kü bel C, Müllen K (1997). Nanosized polyphenylene dendrimers based upon pentaphenylbenzene units. J Mater Chem.

[CR62] Moritz R, Zardalidis G, Butt HJ, Wagner M, Müllen K, Floudas G (2014). Ion size approaching the Bjerrum length in solvents of low polarity by dendritic encapsulation. Macromolecules.

[CR63] Muller S., Mullen K. (2007). Expanding benzene to giant graphenes: towards molecular devices. Philosophical Transactions of the Royal Society A: Mathematical, Physical and Engineering Sciences.

[CR64] Narita A, Feng X, Hernandez Y, Jensen SA, Bonn M, Yang H, Verzhbitskiy IA, Casiraghi C, Hansen MR, Koch AHR, Fytas G, Ivasenko O, Li B, Mali KS, Balandina T, Mahesh S, de Feyter S, Müllen K (2013). Synthesis of structurally well-defined and liquid-phase-processable graphene nanoribbons. Nat Chem.

[CR65] Narita A, Wang X-Y, Feng X, Müllen K (2015). New advances in nanographene chemistry. Chem Soc Rev.

[CR66] Newkome GR, Shreiner CD (2008). Poly (amidoamine), polypropylenimine, and related dendrimers and dendrons possessing different 1/2 branching motifs: an overview of the divergent procedures. Polymers Aligned Carbon Nanotubes: Active Composite Materials.

[CR67] Nguyen TT, Turp D, Wang D, Noscher B, Laquai F, Mullen K (2011). A fluorescent, shape-persistent dendritic host with photoswitchable guest encapsulation and intramolecular energy transfer. J Am Chem Soc.

[CR68] Nguyen TTT, Baumgarten M, Rouhanipour A, Räder HJ, Lieberwirth I, Müllen K (2013). Extending the limits of precision polymer synthesis: giant polyphenylene dendrimers in the megadalton mass range approaching structural perfection. J Am Chem Soc.

[CR69] Nguyen TTT, Türp D, Wagner M, Müllen K (2013). Photoswitchable conductivity in a rigidly dendronized salt. Angewandte Chemie Int Edition..

[CR70] Ornelas Catia, Ruiz Jaime, Belin Colette, Astruc Didier (2009). Giant Dendritic Molecular Electrochrome Batteries with Ferrocenyl and Pentamethylferrocenyl Termini. Journal of the American Chemical Society.

[CR71] Petersen JF, Tortzen CG, Pittelkow M, Christensen JB (2015). Synthesis and properties of chiral internally branched. Tetrahedron.

[CR72] Pisula W, Kastler M, Yang C, Enkelmann V, Müllen K (2007). Columnar mesophase formation of cyclohexa-m-phenylene-based macrocycles. Chem Asian J.

[CR73] Pötzsch R, Voit B (2012). Thermal and photochemical crosslinking of hyperbranched polyphenylene with organic azides. Macromol Rapid Commun.

[CR74] Qin T, Ding J, Wang L, Baumgarten M, Zhou G, Müllen K (2009). A divergent synthesis of very large polyphenylene dendrimers with iridium (III) cores: molecular size effect on the performance of phosphorescent organic light-emitting diodes. J Am Chem Soc.

[CR75] Qin T, Ding J, Baumgarten M, Wang L, Müllen K (2012). Red-emitting dendritic iridium (III) complexes for solution processable phosphorescent organic light-emitting diodes. Macromol Rapid Commun.

[CR76] Rao D, Lu R, Meng Z, Wang Y, Lu Z, Liu Y, Chen X, Kan E, Xiao C, Deng K, Wu H (2014). Electronic properties and hydrogen storage application of designed porous nanotubes from a polyphenylene network. Int J Hydrog Energy.

[CR77] Rosenfeldt S, Dingenouts N, Pötschke D, Ballauff M, Berresheim AJ, Müllen K, Lindner P, Saalwächter K (2005). Analysis of the spatial structure of rigid polyphenylene dendrimers by small-angle neutron scattering. J Lumin.

[CR78] Ruffieux P, Wang S, Yang B, Sanchez-Sanchez C, Liu J, Dienel T (2016). On-surface synthesis of graphene nanoribbons with zigzag edge topology. Nature.

[CR79] Ruiz Jaime, Lafuente Gustavo, Marcen Sylvia, Ornelas Catia, Lazare Sylvain, Cloutet Eric, Blais Jean-Claude, Astruc Didier (2003). Construction of Giant Dendrimers Using a Tripodal Building Block. Journal of the American Chemical Society.

[CR80] Schlupp M, Weil T, Berresheim AJ, Wiesler UM, Bargon J, Mllen K (2001). Polyphenylene dendrimers as sensitive and selective sensor layers. Angewandte Chemie - Int Edition..

[CR81] Schlütter F, Nishiuchi T, Enkelmann V, Müllen K (2014). Octafunctionalized biphenylenes: molecular precursors for isomeric graphene nanostructures. Angewandte Chemie - Int Edition..

[CR82] Shen X, Ho DM, Pascal RA (2004). Synthesis of polyphenylene dendrimers related to “cubic graphite”. J Am Chem Soc.

[CR83] Slota Michael, Keerthi Ashok, Myers William K., Tretyakov Evgeny, Baumgarten Martin, Ardavan Arzhang, Sadeghi Hatef, Lambert Colin J., Narita Akimitsu, Müllen Klaus, Bogani Lapo (2018). Magnetic edge states and coherent manipulation of graphene nanoribbons. Nature.

[CR84] Stangenberg R, Saeed I, Kuan SL, Baumgarten M, Weil T, Klapper M, Müllen K (2014). Tuning polarity of polyphenylene dendrimers by patched surface amphiphilicity—precise control over size, shape, and polarity. Macromol Rapid Commun.

[CR85] Stangenberg R, Türp D, Müllen K (2014). Shape persistent hybrid dendrimers from benzene and triazole via “click chemistry.”. Tetrahedron.

[CR86] Stangenberg R, Wu Y, Hedrich J, Kurzbach D, Wehner D, Weidinger G, Kuan SL, Jansen MI, Jelezko F, Luhmann HJ, Hinderberger D, Weil T, Müllen K (2015). A polyphenylene dendrimer drug transporter with precisely positioned amphiphilic surface patches. Advanced Healthcare Materials.

[CR87] Sun Q, Zhang C, Li Z, Kong H, Tan Q, Hu A, Xu W (2013). On-surface formation of one-dimensional polyphenylene through Bergman cyclization. J Am Chem Soc.

[CR88] Tan YZ, Osella S, Liu Y, Yang B, Beljonne D, Feng X, Müllen K (2015). Sulfur-annulated hexa-peri-hexabenzocoronene decorated with phenylthio groups at the periphery. Angewandte Chemie Int Edition..

[CR89] Taubert A, Wiesler UM, Mullen K (2003). Dendrimer-controlled one-pot synthesis of gold nanoparticles with a bimodal size distribution and their self-assembly in the solid state. J Mater Chem.

[CR90] Tomalia Donald A., Hall M., Hedstrand David M. (1987). Starburst dendrimers. III. The importance of branch junction symmetry in the development of topological shell molecules. Journal of the American Chemical Society.

[CR91] Tomalia, B. D. A., Naylor, A. M., & Goddard, W. A. (1990). Starburst dendrimers: molecular-level control of size, shape, surface chemistry, topology, and flexibility from atoms to macroscopic matter **, *29*, 138–175

[CR92] Türp D, Wagner M, Enkelmann V, Müllen K (2011). Synthesis of nanometer-sized, rigid, and hydrophobic anions. Angewandte Chemie Int Edition..

[CR93] Türp D, Nguyen T-T-T, Baumgarten M, Müllen K (2012). Uniquely versatile: nano-site defined materials based on polyphenylene dendrimers. New J Chem.

[CR94] Walther A, Müller AHE (2013). Janus particles: synthesis, self-assembly, physical properties, and applications. Chem Rev.

[CR95] Water-soluble hyperbranched polyphenylene. (n.d.)

[CR96] Watson MD, Fechtenkötter A, Müllen K (2001). Big is beautiful—“aromaticity” revisited from the viewpoint of macromolecular and supramolecular benzene chemistry. Chem Rev.

[CR97] Waybright SM, McAlpine K, Laskoski M, Smith MD, Bunz UHF (2002). Organometallic dendrimers based on (tetraphenylcyclobutadiene) cyclopentadienylcobalt modules. J Am Chem Soc.

[CR98] Weil T, Wiesler UM, Herrmann A, Bauer R, Hofkens J, de Schryver FC, Müllen K (2001). Polyphenylene dendrimers with different fluorescent chromophores asymmetrically distributed at the periphery. J Am Chem Soc.

[CR99] Wiesler, U.-M., & Müllen, K. (1999). Polyphenylene dendrimers via Diels–Alder reactions: the convergent approach. 10.1039/A907339F

[CR100] Wiesler UM, Berresheim AJ, Morgenroth F, Lieser G, Müllen K (2001). Divergent synthesis of polyphenylene dendrimers: the role of core and branching reagents upon size and shape. Macromolecules.

[CR101] Wind, M., Wiesler, U.-M., Saalwächter, K., Müllen, K., & Spiess, H. W. (2000). Shape-Persistent Polyphenylene DendrimersÐ Restricted Molecular Dynamics from Advanced Solid-State Nuclear Magnetic Resonance Techniques**. *Appl. Phys. Lett. Appl. Phys. Lett. Macromolecules*, *761718192021*(1)

[CR102] Wind M, Saalwächter K, Wiesler UM, Müllen K, Spiess HW (2002). Solid-state NMR investigations of molecular dynamics in polyphenylene dendrimers: evidence of dense-shell packing. Macromolecules.

[CR103] Wu J, Gherghel L, Watson MD, Li J, Wang Z, Simpson CD, Kolb U, Müllen K (2003). From branched polyphenylenes to graphite ribbons. Macromolecules.

[CR104] Wu Jishan, Grimsdale Andrew C., Müllen Klaus (2005). Combining one-, two- and three-dimensional polyphenylene nanostructures. J. Mater. Chem..

[CR105] Yang X, Dou X, Müllen K (2008). Efficient synthesis of symmetrically and unsymmetrically substituted hexaphenylbenzene analogues by Suzuki-Miyaura coupling reactions. Chem Asian J.

[CR106] Yin M, Bauer R, Klapper M, Müllen K (2007). Amphiphilic multicore-shell particles based on polyphenylene dendrimers. Macromol Chem Phys.

[CR107] Zhang H, Grim PCM, Foubert P, Vosch T, Vanoppen P, Wiesler UM (2000). Properties of single dendrimer molecules studied by atomic force microscopy. Langmuir.

[CR108] Zhang Dengke, Yao Huajie, Zhou Dongju, Dai Libo, Zhang Jie, Yuan Siguo (2014). Synthesis, characteristics and adsorption properties of polyphenylene sulfide based strong acid ion exchange fiber. Polymers for Advanced Technologies.

[CR109] Zhang G, Baumgarten M, Auer M, Trattnig R, List-Kratochvil EJW, Mullen K (2014). Core-and-surface-functionalized polyphenylene dendrimers for solution-processed, pure-blue light-emitting diodes through surface-to-core energy transfer. Macromol Rapid Commun.

[CR110] Zhi L, Wu J, Li J, Stepputat M, Kolb U, Müllen K (2005). Diels-Alder reactions of tetraphenylcyclopentadienones in nanochannels: fabrication of nanotubes from hyperbranched polyphenylenes. Adv Mater.

[CR111] Zhi L, Wang J, Cui G, Kastler M, Schmaltz B, Kolb U, Jonas U, Müllen K (2007). From well-defined carbon-rich precursors to monodisperse carbon particles with hierarchic structures. Adv Mater.

[CR112] Zöphel L, Berger R, Gao P, Enkelmann V, Baumgarten M, Wagner M, Müllen K (2013). Toward the peri-pentacene framework. Chem Eur J.

